# Cerebellar ependymal cyst: a case report

**DOI:** 10.3389/fnins.2024.1372410

**Published:** 2024-04-03

**Authors:** Chengye Hou, Yuanqin Liu, Feng Li, Qinglu Zhang

**Affiliations:** ^1^Department of Neurosurgery, The First Affiliated Hospital of Shandong First Medical University & Shandong Provincial Qianfoshan Hospital, Shandong Medicine and Health Key Laboratory of Neurosurgery, Jinan, China; ^2^Department of Ultrasound Medicine, Shandong Provinicial Third Hospital, Shandong University, Jinan, Shandong, China

**Keywords:** ependymal cyst, cerebellum, cerebral aqueduct, fourth ventricle, glial fibrillary acidic portein (GFAP)

## Abstract

**Rationale:**

Intracranial ependymal cysts are relatively rare. The current case report focuses on a patient who was diagnosed with an ependymal cyst and underwent surgical treatment. Postoperative pathological examination confirmed the presence of this lesion in the cerebellum.

**Chief complaint:**

A 32-year-old female patient presented with a chief complaint of dizziness and headache with no triggers for the past 1 year. She also reported an increase in the frequency and intensity of symptoms in the past 2 weeks.

**Diagnosis:**

Cranial magnetic resonance imaging (MRI) showed a rounded long T1 and T2 abnormal signal foci in the left posterior part of the brainstem under the cerebellar pallidum. The lesion had a clear boundary, was approximately 4.0 × 3.1 × 3.2 cm in size, and did not exhibit any definitive enhancement.

**Interventions:**

Total resection of the lesion was carried out after completion of the preoperative examination.

Treatment outcomes. The patient was discharged from the hospital on postoperative day 11 once their symptoms had disappeared. The sensory and motor functions of the limbs remained unaffected by treatment.

**Experiences:**

Cerebellum ependymal cysts are rare, and most patients only experience discomfort due to cerebral edema. These lesions are also often difficult to differentiate from other intracranial cysts using imaging alone. The aim of this study was to report a rare case of ependymal cyst so that it may serve as a reference for diagnosis and treatment in the future.

## Introduction

Ependymal cysts are rare, benign neuroepithelial lesions that primarily occur in the brain parenchyma but can also be found in the subarachnoid space, spinal canal, brainstem, pontine cerebellar angle, and rarely in the ventricle of the brain (Boockvar et al., 2000). The majority of cysts are clinically asymptomatic and are detected during routine imaging. However, patients may exhibit symptoms upon excessive cyst enlargement that causes compression of the brain parenchyma and affects cerebrospinal fluid circulation. The extent of symptoms can vary with the size and location of the lesion. In such patients, surgical intervention may be necessary to reduce hydrocephalus (Ho and Wu, 2009). Currently, there is limited research on ependymal cysts, they can be difficult to differentiate from other benign intracranial lesions by imaging alone, so they are often misdiagnosed (Ciricillo et al., 1990). Therefore, the final diagnosis should only be made after observation of a cystic lesion using imaging, histopathological confirmation of an ependymal cyst postoperatively, and positive glial fibrillary acidic portein (GFAP) staining using immunohistochemical analysis (Inoue et al., 1988).

## Clinical presentation

### Patient characteristics

The current case report focuses on a female patient (age 32 years) who had a chief complaint of bouts of dizziness and headaches for a period of 1 year, with increasing frequency and intensity in the past 2 weeks. No vomiting, confusion, inability to speak, or mental/behavioral abnormalities were observed in association with the headaches.

Physical examination showed normal vision in both eyes, no memory loss, no visual field defects, normal deep and superficial sensation in the trunk and limbs, normal movement of the limbs, and negative pathological signs. Cranial magnetic resonance imaging (MRI) showed a rounded long T1 and T2 abnormal signal foci in the left posterior part of the brainstem inferior to the cerebellar vermis; low signals in the T2-FLAIR and DWI; a well-defined lesion border; a lesion size of approximately 4.0 × 3.1 × 3.2 cm; and no definite enhancement. The cyst was seen to be pushing into the surrounding brain tissue, midbrain aqueduct, and fourth ventricle, causing changes in these areas. Moreover, the supratentorial ventricle was seen to be dilated; strips of long T2 and high T2-FLAIR signals were observed bilaterally around the lateral ventricles; the gyri and sulci did not appear wider or deeper; and the midline structure was seen to be centered.

### Treatment

Surgical intervention using the left occipital approach was considered to be the most suitable treatment option based on the findings of the imaging examination. Dural exposure revealed slightly high cerebral pressure and no defects or atrophy in the cerebral cortex except for slight swelling. Thereafter, decompression was achieved by releasing cerebrospinal fluid from the occipital pool; the cystic wall was exposed by probing 2 cm deeper into the incisural space Then punctured the cystic wall to release a colorless clear fluid. Further retraction exposed the complete cyst envelope and the dorsal surface of the brainstem in the deeper portion of the cyst. The lesion tissue samples were carefully peeled off and collected for pathological examination. Thereafter, artificial dural material was tightly sutured to the dura mater after confirming that cerebrospinal fluid circulation is unblocked and adequate hemostasis.

Postoperative pathological and immunohistochemical examination showed that the tissue specimens were soft, grayish-red in color, while light microscopy examination of the cyst wall confirmed the presence of columnar epithelial cells exhibiting ciliated structures on the surface, absence of a distinct basement membrane, direct attachment to the glial cells, and lack of plexiform organization. Immunohistochemical examination showed positive GFAP, negative CK, and approximately 3% Ki-67.

### Postoperative pathological section of cyst

Postoperative follow-up showed elevated body temperature (highest value: 37.9°C) on days 2 and 3 and a gradual decrease in symptoms (i.e., headache and dizziness) from day 7. During the patient’s febrile period, the patient underwent a lumbar puncture and other relevant laboratory tests, and no abnormalities were found on these tests. The increase in body temperature was considered to be a normal bodily reaction to the absorption of the cystic fluid. All symptoms were seen to disappear after the reducing cranial pressure treatment and one week of anti-inflammatory treatment. At the time of discharge from the hospital on day 11, the patient was in a stable condition without postoperative complications. Moreover, no evidence of recurrence was seen during the one-year follow-up period. See Supplementary Figure 1 for MRI images of the patient. See Supplementary Figure 2 for light microscopic pathology images of the patient.

## Discussion

The majority of patients exhibit good outcomes following treatment of benign ependymal cysts, with only one study to date reporting patient death, cause of death is the neurosurgical procedure itself (Paulla Galdino Chaves et al., 2022). Although the origins of ependymal cysts remain unclear, it has been proposed that they may arise from ectopic displacement of ventricular cells during embryonic development (Friede and Yasargil, 1977). During embryonic development, the abnormal ependymal cysts in the primitive rhombencephalic vermis expand into the brain parenchyma and then gradually enlarge as the patient grows, eventually compressing the brain parenchyma or affecting cerebrospinal fluid circulation, and leading to the development of the corresponding symptoms (Sharpe and Deck, 1977). Headache is the most common clinical manifestation of ependymal cysts. Oversized cysts often lead to obstruction of cerebrospinal fluid circulation, and some patients may show symptoms of obstructive hydrocephalus, such as headache, nausea, vomiting, blurred vision, drowsiness, and seizures (Czervionke et al., 1987). The development process of these lesions is biased towards a benign progression, before cystic lesions were discovered during casual imaging, a number of patients didn’t perceive the presence of early lesion, which has led to a minimal number of reports about them in the literature, and there is less research in this field can also be attributed to this. Data on past patients in the literature can be found in Supplementary Table 1 (van Lindert et al., 1998; Shin et al., 2000; Roberti and Magram, 2005; Kumar et al., 2006; Endo et al., 2009; Fonoff et al., 2010).

Differentiation of ependymal cysts from other benign cranial lesions such as arachnoid, endothelioid, or endothelial cysts can be difficult using imaging alone (Ciricillo et al., 1990). Computed tomography (CT) scans show that these round lesions are similar in density to cerebrospinal fluid, and the MRI imaging of the fluid in cysts shows signals similar to cerebrospinal fluid-like signal. This can also be used to rule out the presence of other lesions, such as gliosis or soft tissue masses.

Due to the rarity of these lesions, no standardized treatment protocols have been developed to date. Surgical intervention is deemed necessary in patients presenting with symptoms such as headaches and blurred vision, with the most common surgical approaches include being open total resection, partial resection communicating ventricular shunts, cyst deroofing and opening, and stereotactic suction (El Damaty et al., 2017). The surgical approach of choice varies with the location of the cyst, and some articles have suggested the use of neuronavigation for intraoperative localization of the lesion in order to minimize the damage to the brain parenchyma (Prieto et al., 2013). These treatment measures typically aim to relieve compression of the brain tissue and restore cerebrospinal fluid circulation, with the majority of cases involving the removal of the capsule wall and connection of the capsule cavity to the ventricular system. This approach reduces brain tissue damage during total cystectomy while also allowing the cystic fluid to be recycled and metabolized through the ventricular system, preventing postoperative lesion enlargement. However, some patients have experienced cyst recurrence, after undergoing partial resection communicating ventricular shunts or stereotactic suction. To avoid this, a concomitant ventriculoperitoneal shunt is recommended for patients with poor connection of the capsule chambers to the ventricular system. Most patients exhibit improvement in symptoms after surgery, with only one patient dying due to surgical complications to date (Paulla Galdino Chaves et al., 2022). However, the recurrence rates of these cysts remain unknown due to limited long-term follow-up evidence.

Ependymal cysts have a translucent or light gray membrane, do not communicate with the ventricular system or the subarachnoid space, and contain a clear fluid (similar to cerebrospinal fluid) that is actively secreted by the epithelial cells leading to progressive growth of the lesion and gradual worsening of symptoms such as intracranial hypertension and cerebral edema (Boockvar et al., 2000). Histopathological examination shows that the cystic wall is divided into the inner and outer layers, with the former consisting of a single layer of cylindrical cells (similar to ventricular cells) containing ciliated structures and the latter directly connecting to the brain tissue or glial cells (Friede and Yasargil, 1977). Immunohistochemical staining shows positive GFAP, which, in conjunction with the imaging findings, allows definitive diagnosis.

## Conclusion

Ependymal cysts in the cerebellum are clinically rare and difficult to distinguish from other benign neurological cysts. Therefore, the diagnosis should be made taking clinical signs, imaging findings, and positive immunohistochemical GFAP into consideration. The appropriate surgical approach should be selected based on the location of the lesion and its adjacent structures. Future studies should aim to carry out long-term follow-up of these patients to estimate recurrence rates at a later stage.

## Data availability statement

The original contributions presented in this study are included in this article/Supplementary material, further inquiries can be directed to the corresponding authors.

## Ethics statement

Written informed consent was obtained from the participant/patient(s) for the publication of this case report.

## Author contributions

CH: Data curation, Writing – original draft, Writing – review and editing. YL: Conceptualization, Supervision, Writing – review and editing. FL: Resources, Writing – review and editing. QZ: Writing – review and editing.

## Supplementary material

Supplementary Figure 1 **(a)** Preoperative,MRI,T1W. **(b)** Preoperative,MRI,T1W,Sagittal fault. **(c)** Preoperative,MRI,T1W,Coronal fault. **(d)** Preoperative,MRI,T1W,Enhancement scan. **(e)** Preoperative,MRI,T2W. **(f)** Postoperative,MRI,T1W. **(g)** Postoperative,MRI,T1W,Enhancement scan. **(h)** Postoperative,MRI,T2W.

Supplementary Figure 2 Shows the patient’s postoperative pathology under light microscopy.

Supplementary Table 1 Shows the data’s of patients in some of the past literature.
