# Supplementary figures and images for: Cerebellar ependymal cyst: a case report
Source: Front Neurosci. 2024 Apr 3;18:1372410. doi: 10.3389/fnins.2024.1372410 (PMC11022958; doi:10.3389/fnins.2024.1372410)

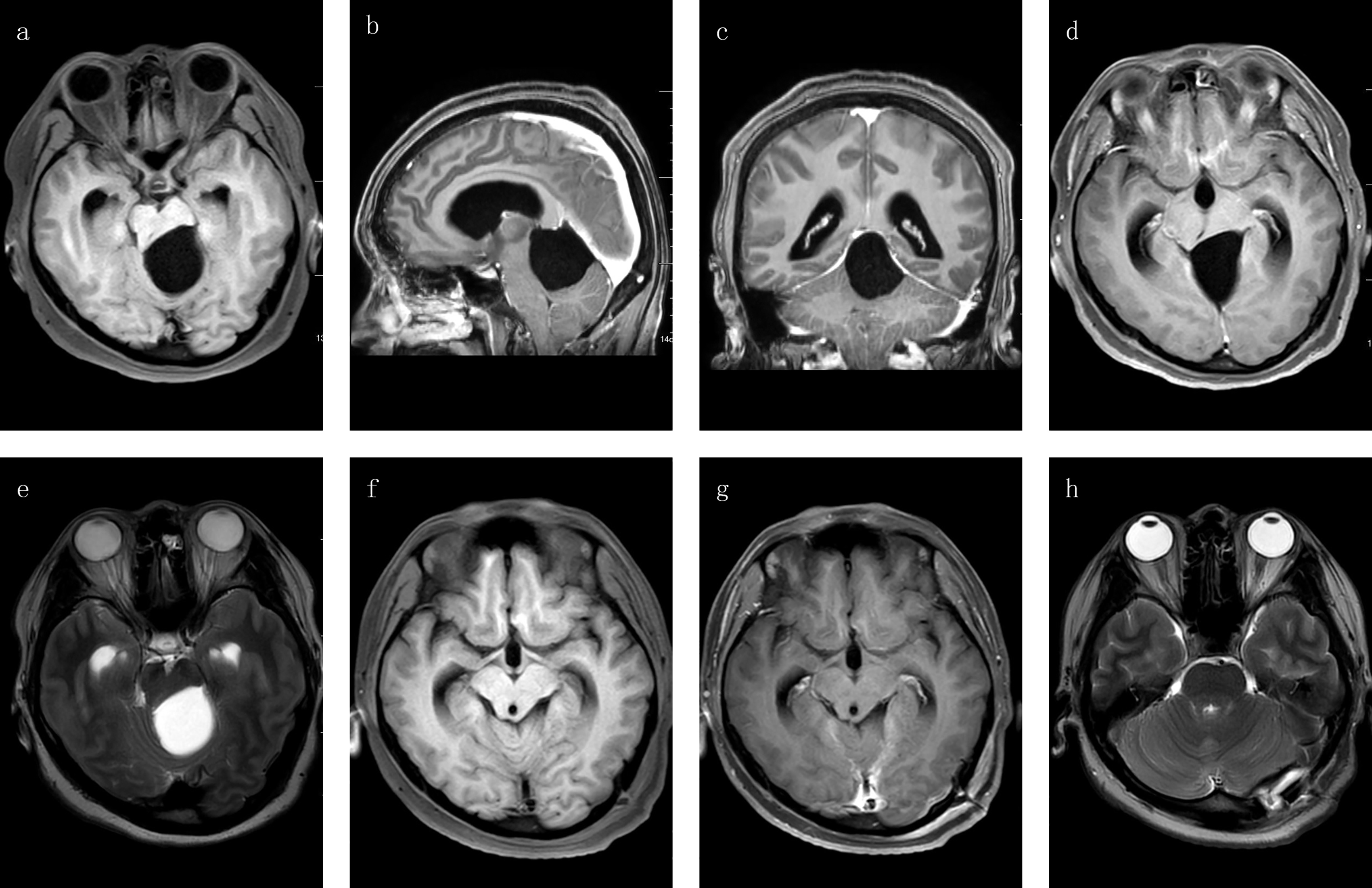

Supplement: Supplementary Figure 1 — (a) Preoperative,MRI,T1W. (b) Preoperative,MRI,T1W,Sagittal fault. (c) Preoperative,MRI,T1W,Coronal fault. (d) Preoperative,MRI,T1W,Enhancement scan. (e) Preoperative,MRI,T2W. (f) Postoperative,MRI,T1W. (g) Postoperative,MRI,T1W,Enhancement scan. (h) Postoperative,MRI,T2W. [file Image_1.JPEG]

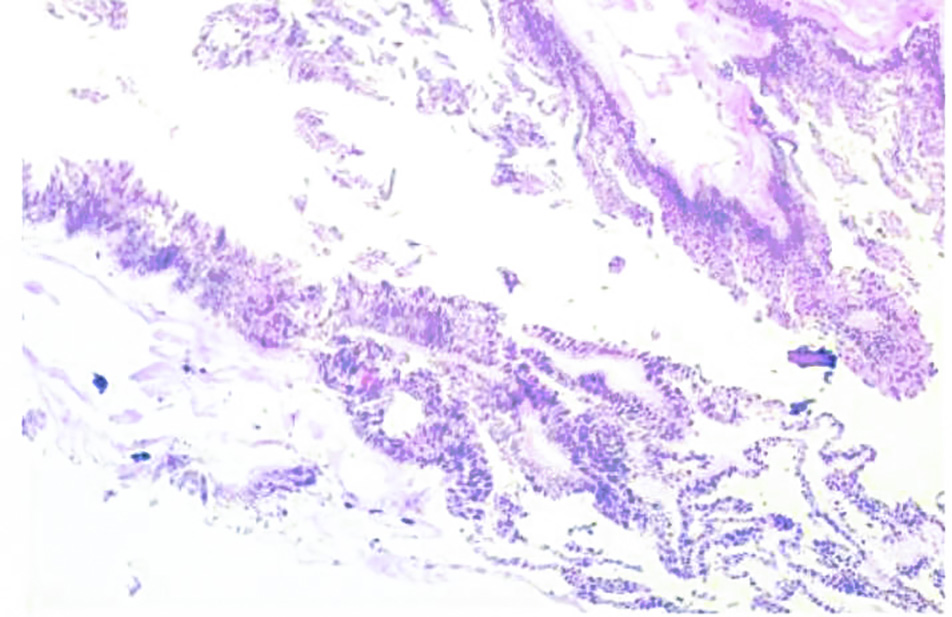

Supplement: Supplementary Figure 2 — Shows the patient’s postoperative pathology under light microscopy. [file Image_2.JPEG]
